# Progress in the synthesis of imide-based N-type polymer semiconductor materials

**DOI:** 10.1039/d0ra04972g

**Published:** 2020-11-17

**Authors:** Mao Liao, Jieming Duan, Peng'ao Peng, Jingfeng Zhang, Ming Zhou

**Affiliations:** School of New Energy and Material, Southwest Petroleum University No. 8 Xindu Avenue, Xindu District Chengdu Sichuan 610500 People's Republic of China 1902571422@qq.com 86453501@qq.com 939539132@qq.com 865387887@qq.com drzhouming@sohu.com +8613880947076; State Key Laboratory of Oil and Gas Reservoir Geology and Exploitation, Southwest Petroleum University No. 8 Xindu Avenue, Xindu District Chengdu Sichuan 610500 People's Republic of China; CNBM (Chengdu) Optoelectronic Materials Co., Ltd. No. 558, 2nd Airport Road, Shuangliu District Chengdu Sichuan 610207 People's Republic of China

## Abstract

Based on the development situation and challenge of organic photovoltaics (OPVs) and organic field-effect transistors (OFETs), it is necessary to develop N-type polymer building blocks with specific structures and performance. After decades of development, some excellent polymer receptor building blocks have been developed to construct N-type organic semiconductors, which have been applied in OFETs and OPVs. In this paper, four kinds of imide (bisthiophene imide BTI, bisthiazolimide BTz, naphthalimide NDI, and perylene imide PDI)-based N-type polymer semiconductor materials are introduced, and their applications in OFETs and OPVs are analyzed, too. The molecular structure design and the performance of corresponding materials are summarized to provide further guidance and reference for the design and development of high performance N-type polymer semiconductors.

## Abstract 2

Representative molecular structures of four N-type polymer semiconductors materials (a: N2000; b: PPDI-DTT, c: TBDI-DT and d: PDTzTIT) based on NDI, PDI, BTI and BTzI, respectively.

## Graphical abstract

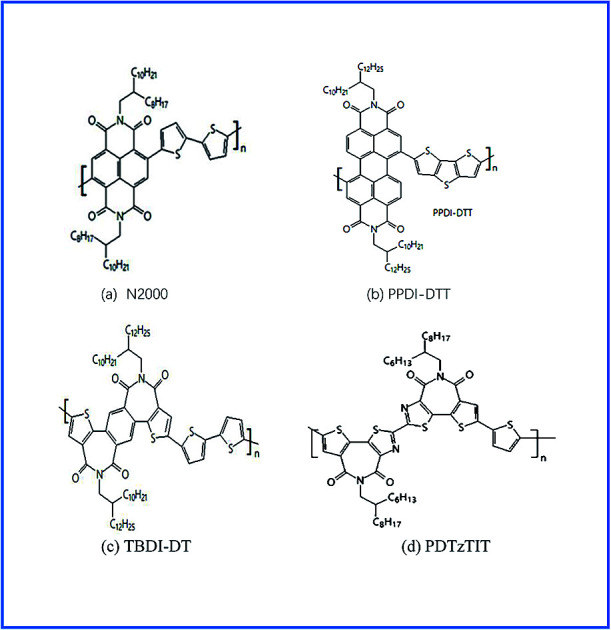

## 1 Introduction

In 1977, H. Shirakawa, M. MacDiarmid and A. J. Heeger discovered the conductive polyacetylene materials for the first time,^1^ which overturned people's understanding of the non-conductivity of organic materials and opened up the field of organic electronics. This major discovery was awarded the Nobel Prize in chemistry in 2000. In the 1980s, organic semiconductor materials were gradually applied to the OFETs and OPVs fields. Ando *et al.*^2^ prepared the first field-effect transistor by using organic macromolecular polythiophene films as semiconductors. Tang^3^ prepared a thin-film two-layer organic photovoltaic cell using copper phthalocyanine and perylene tetracarboxylic acid derivatives as raw materials. Compared with conventional transistors, OFETs has advantages such as simple preparation process, low cost, light weight and flexibility, but its general low charge capture ability limits its commercial feasibility. Because organic semiconductors are the core components of OFETs, the development of OFETs depends on the development of organic semiconductors to a great extent. In the past decades, fullerenes have been used as the receptor unit for most OPVs. However, fullerenes have the disadvantages of weak solar absorption, difficulty in energy level regulation, high production cost and poor morphology stability, which limit the sustainable development of OPVs. Later, breakthrough research in the field of organic semiconductors provided ideas to solve these problems.

Polymer semiconductor materials not only have electronic properties of metals or semiconductors, but also have excellent processing properties and mechanical properties. Organic semiconductor materials have good solubility by introducing side chain, which makes it easy to be processed into solubilized or slurried electronic inks. In the fabrication process of OFETs, spin coating technology or ink-jet printing technology can be used to prepare organic semiconductor thin films, so as to realize the preparation of large-area and low-cost organic optoelectronic devices and circuits at room temperature and atmospheric pressure without expensive methods such as high-temperature heating, electroplating and evaporation, which not only simplifies the production process, but also reduces the processing difficulty and production cost. Polymer semiconductor materials have many advantages such as simple preparation process, low cost, light weight, flexibility, transparency, portability and broad application prospects, which have become a research hotspots in recent years.^4–13^ Due to these unique advantages, the researches of optoelectronic polymer materials have attracted extensive attention of academic and industrial circles at home and abroad, and optoelectronic polymer materials and their applications in related optoelectronic devices have also experienced rapid development. Organic semiconductors can be divided into three categories according to the carrier transport types: (1) P-type polymer semiconductors, the hole transport capacity is significantly better than the electron transport capacity; (2) N-type polymer semiconductors, the electron transport capacity is significantly better than the hole transport capacity; (3) bipolar polymer semiconductors, hole and electron transport capacity is equivalent. P-type polymer semiconductor materials are also known as hole transport materials, in which pentacene, polythiophene, diketopyrrolopyrrole (DPP) and isoindigo (IDG) derivatives are represented. N-type polymer semiconductor materials are also known as electron transport materials. When the energy level difference between donor and acceptor is greater than the binding energy of electron, charge transfer excited state is formed. Finally, the separated electrons and holes are transported in the acceptor and donor materials by local state hopping. The classical N-type polymers mainly include imides, amide, B ← N-embedded polymers and cyano polymers.^14–16^

To achieve N-type properties, polymer semiconductors usually need to introduce strong electron groups to reduce the Frontier orbital energy levels of semiconductors. Due to the high steric hindrance, N-type polymer semiconductors decrease the coplanarity and crystallinity of molecules, which is not conducive to carrier transfer, and they are difficult to be synthesized, too. Therefore, compared with P-type polymer semiconductor materials, the researches of n-type polymer semiconductor materials are obviously lagging behind, and the researches of high-performance n-type polymer semiconductor materials are still less, but it is of great significance. As an electron transport materials, the N-type semiconductors are necessary to construct P–N junction with P-type semiconductors, which has great potential in large scale integrated circuit applications. The conjugated skeleton structure of polymer semiconductors largely determines the properties of semiconductor materials and device performance. Therefore, the introduction of strong electron withdrawing groups in the polymer skeleton can optimize the properties of the material, thereby realizing and improving the performance of N-type semiconductors. Compared with other electron withdrawing groups, imide has stronger electron withdrawing ability, which is more conducive to the realization of N-type device performance. The imide group not only has the ability of strong electron withdrawing, but also can obtain good solubility and solution processability by introducing a dissolving chain into the nitrogen atom. In terms of transistor applications, as the active layer material, the strong electron withdrawing ability of imide can effectively reduce the orbital energy level of the polymer, so that electrons can be effectively injected and stabilize device performance in the air. In the application of solar cells, as the acceptor materials, in order to enhance the ability to capture light, and to absorb and complement with the wide band gap donor unit as far as possible, it can condense the imide and extend the conjugated skeleton structure to promote the polymer to absorb more red shift and increase the current. This design makes imide based polymer semiconductor have better application prospect in the field of organic solar cells. Therefore, the design and development of new high-performance N-type polymer semiconductor materials will provide a great impetus to the development of organic electronics.^17–20^

The latest researches progress of N-type polymer semiconductor materials and their device performance in OFETs and OPVs are introduced in this paper. The synthesis and device application of N-type polymer semiconductor materials based on imides are emphatically expounded. The structure–property relationship of materials in different devices (OFETs and OPVs) is reviewed. Finally, some opinions on further material research are provided based on the experience in the researches of N-type polymer semiconductor materials.

## 2 Preparation methods of imide-based N-type polymer semiconductor materials

N-Type polymer semiconductor materials can be used as electron transport materials in many different semiconductor devices. Different semiconductor devices have different requirements for their material properties. Generally speaking, N-type polymer semiconductor materials should have lower LUMO (lowest unoccupied molecular orbital) levels to ensure carrier electron injection (or generation).).^12^ Because of the high LUMO level of most electronic transport materials, the organic anions of electron carriers are prone to react with water and oxygen in the air. As a result, electrons are captured, which reduces the performance and stability of the device performance of N-type polymer semiconductors in the air. Therefore, it is necessary to test and operate in the vacuum or nitrogen atmosphere. It is often believed that when the LUMO level of organic semiconductors is lower than −4.0 eV, the generated organic carbon anions can exist stably in the air and form stable electron transport. Therefore, how to effectively reduce the LUMO level of semiconductor materials is the key to the design of the high performance N-type polymer semiconductors. The coplanarity of the main chain, the stacking and alignment of molecules in the solid-state, and the crystallinity of materials play a key role in carrier transport, which largely determines the performance of devices. The polymer skeleton structure with good coplanar properties can strengthen the orbital overlap and π–π stacking between molecular chains, which is conducive to the self-assembly of long-process well-ordered polymer polycrystalline films. The solubility, molecular weight and aggregation structure of the polymer can be controlled by designing the type, length and branching point of the soluble alkyl chain, so as to improve the charge transfer performance. Therefore, good co-planarity and symmetry, excellent solubility, high crystallinity and compact and ordered packing arrangement can obtain excellent polymer semiconductors.

The preparation methods for constructing N-type polymer semiconductor materials was analyzed in detail, which included researches and development of electron-deficient construction units (receptor units), and introduction of electron-withdrawing groups. After introducing electron-deficient construction units (acceptor units) into polymer skeleton, the LUMO level of polymer semiconductor materials can be effectively reduced due to the strong electron-withdrawing ability of electron-deficient units, which is conducive to the realization of N-type properties.^21^ The design and synthesis of electron-deficient units is the most important part of the development of high-performance N-type polymer semiconductor materials. At present, the main electron-deficient units are fused-ring electron acceptors, which mainly include: imide construction units (naphthalene diimide NDI, perylene diamide PDI, bisthiophene imide BTI, bisthiazole imide BTzI, *etc.*),^22–32^ B–N bond construction units,^33,34^ benzothiadiazole (BT) construction units.^35–37^ Compared with other groups, imide group has the following advantages: (1) imide group has strong electron withdrawing effect, which can effectively reduce the LUMO energy level of semiconductor materials; (2) the electron deficient property of imide monomer and the solubilization of imide side chain are conducive to obtain high molecular weight of polymerization; (3) close molecular spacing is obtained by strong intermolecular interaction; (4) the side chain of imide is beneficial to the self-assembly of polymer and the crystallinity of the material; (5) the carbonyl in the imide is conducive to the formation of intramolecular sulfur⋯oxygen non-covalent bond or hydrogen bond formation,^38–40^ which makes the main chain coplanar. Therefore, imide group is widely used in the construction of high performance N-type polymers.

The imide-based N-type polymer semiconductor materials are introduced in four categories: naphthalene diimide(NDI), perylene diimide (PDI), bisthiophene imide (BTI) and bisthiazole imide (BTzI) as follows. Some of the most important parameters of highlighted n-type polymer semiconductors are summarized in Table 1.

**Table 1:** Performance parameters of highlighted n-type polymer semiconductors

N-Type polymer semiconductors	HOMO/eV	LUMO/eV	*μ*_electron_/(cm^2^ V^−1^ s^−1^)	PCE/%
N2200	−5.86	−4.01	0.85	—
PNDIF-T2	−5.62	−4.01	6.5	—
PNBTDIs	−5.73	−4.0	1.5 × 10^−2^	—
P(PDI-BDT-T)	−5.53	−3.89	—	4.31
f-BTI2-FT	−5.61	−2.86	1.3 × 10^−2^∼4.5 × 10^−2^	—
PBTI1	−5.46	−3.48	3.71	—
s-FBTI2-FT	−5.08	−3.55	2.73	6.50
f-FBTI2-T	−5.94	−3.46	—	8.10
TFBDI-T	−6.01	−3.29	2.1 × 10^−2^	—
PDTzTI	−5.78	−3.77	1.61	—
PTzTIBDTT	−5.65	−3.74	1.1 × 10^−4^	8.00
PTzTIBDTT-S	−5.69	−3.78	1.6 × 10^−5^	3.69

## 3 Preparation of high performance imide N-type polymer semiconductor materials

### 3.1 NDI-based N-type polymer semiconductor materials

**Fig. 1:**
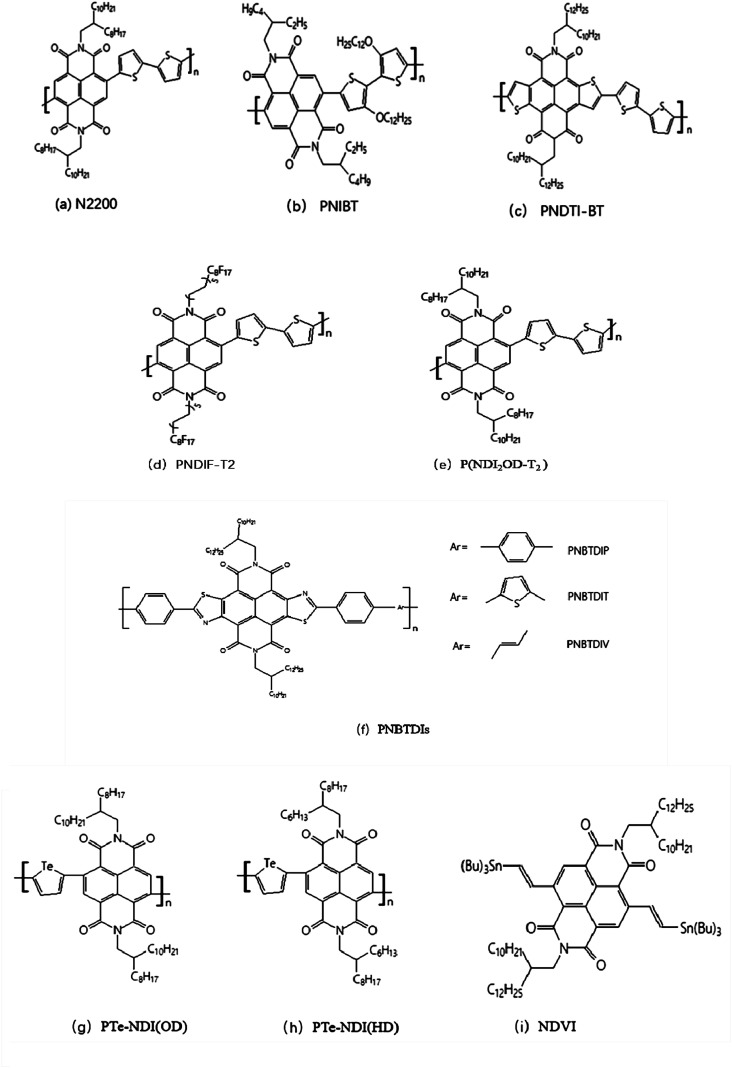
Representative molecular structures (a–i) of naphthalene diimide based N-type polymer semiconductors.

N-Type semiconductor materials of NDI-based polymer N2200, the molecular structure of which was shown in Fig. 1(a), was optimized by Facchetti *et al.* in 2009.^41^ The performances of the top organic field-effect transistor (TOFET) were reported.

In 2010, copolymer PNIBT materials was synthesized by NDI with alkoxy chains modified dithiophene by Watson and Jenekhe.^42^ Copolymer PNIBT in Fig. 1(b) has not only electronics but also hole transport properties which are caused by electron-rich alkoxythiophene. However, there is a certain steric hindrance in NDI unit, which is not conducive to achieving high coplanarity of polymer main chain, thus limiting the improvement of performance of NDI polymer devices to some extent.

In 2013, Takimiya *et al.*^43^ designed and synthesized lacking electronic unit NDTI based on thiophene-modified NDI to reduce the negative effects of NDI steric hindrance. After the two ends of NDI group merge into thiophene ring, the steric resistance of the constructed monomer NDTI decreases dramatically, which greatly improves the planarity of the polymer and enhances the interaction between the corresponding polymer PNDTI-BT molecules, as shown in Fig. 1(c).

Cho *et al.*^44^ developed NDI-based polymer PNDIF-T2 (Fig. 1(d)) with the side chain of semi-fluorination through side-chain optimization in 2016. The results show that the semi-fluorinated side chain can greatly improve the polymer skeleton ordering while maintaining proper solubility, thus achieving electron mobility as high as 6.5 cm^2^ V^−1^ s^−1^ in transistors. In 2019, Wang *et al.*^45^ obtained a new NDVI (Fig. 1(i)) construction block by emerging vinyl into both sides of NDI to reduce the steric hindrance. The new poly(benzothiadiazole-naphthalenediimide) derivatives were prepared by the polymerization of NDVI with benzothiadiazole monomer containing triazole, and its electron mobility is as high as 7.16 cm^2^ V^−1^ s^−1^.

The problem of the morphological control of organic materials in the active layer has been the focus of attention. A lot of researchers had focused on the morphological control of the P-type semiconducting compounds, but the morphological control of the N-type materials had studied infrequently. Di Pietro, *et al.*^46^ studied electron-transporting conjugated polymers P(NDI_2_OD-T_2_) (Fig. 1(e)). The stability of P(NDI_2_OD-T_2_) in oxygen exposure is restricted by the interaction between the neutral polymer and molecular oxygen, which resulted in a reduction in electron mobility in most semiconductors. They used the theory of density functional quantum chemical calculations to ascribe the decrease in mobility to the formation of a shallow, localized, oxygen-induced trap level, 0.34 eV deviated lowest unoccupied molecular orbital below P(NDI_2_OD-T_2_). Conversely, the stability of the polymer anion to water is restricted by two competing reactions, one involving the electrochemical oxidation of polymer anion by water without degrading of the polymer and the other involving the chemical reaction of the polymer catalyzed by a free radical anion with water. The charge can be recycled, which resulted in further degradation reactions. In this way, a major part of the membrane is degraded after a long period of bias stressing. The main reason is the chemical action of the naphthalene diamine unit of polymer and water. The degradation mechanisms should be considered to explain electron capture in other aryl diamines and other conjugated polymers. Yu Jin Kim *et al.*^47^ systematically controlled the morphology of the N-type semiconductor with the introduction to insulating polymers PS and PAN. The different blending ratios of P(NDI_2_OD-T_2_) to PS and P(NDI_2_OD-T_2_) to PAN in film and the structural changes after thermal annealing treatment were analyzed. The results indicated that the molecular morphology of P(NDI_2_OD-T_2_) changes with the random stacking direction. They studied a new method of the morphologically controlled N-type semiconducting polymers, which would benefit to morphological control of other materials.

The New N-type conjugated polymer semiconductors with an electron-deficient naphthobisthiazole diimides (NBTDIs) moiety were synthesized by Selvam.^48^ The optical bandgaps of the PNBTDIs (Fig. 1(f)) thin films are 1.73–2.02 eV at the absorption edge because they have strong absorption band in the visible region. In thin-film transistors, the PNBTDIs exhibit unipolar N-channel transports with electron mobility up to 1.5 × 10^−2^ cm^2^ V^−1^ s^−1^. The results showed that PNBTDIs were a kind of promising material for N-type field-effect transistors and all-polymer solar cells. Novel tellurophene-based N-type copolymers PTe-NDI(OD) and PTe-NDI(HD) (Fig. 1(g)) were synthesized by Lv, L.^49^ The results suggested that PTe-NDI(OD) and PTe-NDI(HD) N-type copolymers were promising electron acceptors for organic solar cells and potential sensor materials for bromine detection.

**Fig. 2:**
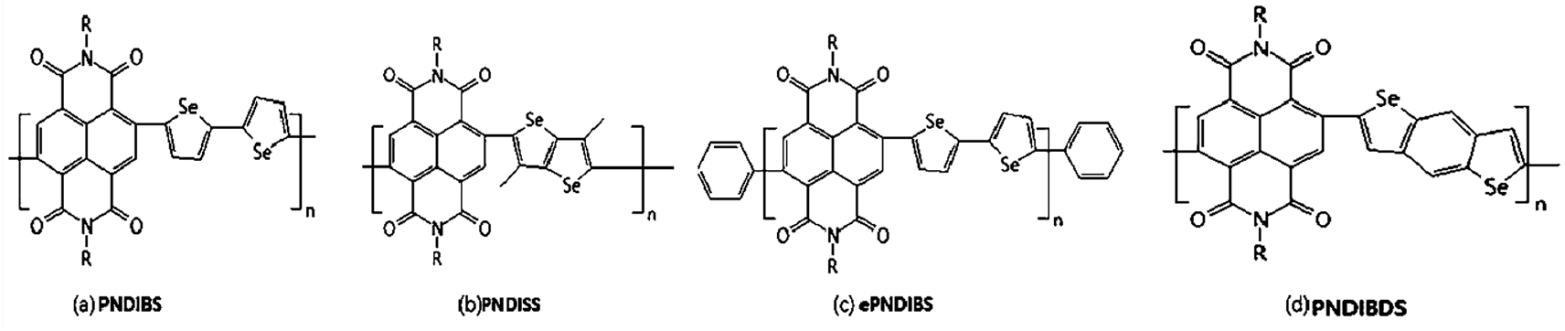
Chemical structures (a–d) of PNDIBS, PNDISS, ePNDIBS and PNDIBDS.

Charge trapping is an undesirable phenomenon and a common challenge in the operation of N-channel organic field-effect transistors. Herein, Nishit M. Murari *et al.*^50^ exploited charge trapping in an N-type semiconducting poly(naphthalene diimide-*alt*-biselenophene) (PNDIBS) (Fig. 2(a)) as the key operational mechanism to develop high performance, nonvolatile, electronic memory devices. The PNDIBS-based field-effect transistor memory devices were programmed at 60 V and they showed excellent charge-trapping and de-trapping characteristics, which could be cycled more than 200 times with a current ratio of 10^3^ between the two binary states. Programmed data could be retained for 10^3^ s with a memory window of 28 V. This is a record performance for N-channel organic transistors with inherent charge-trapping capability without using external charge trapping agents. However, the memory device performance was greatly reduced, as expected, when the N-type polymer semiconductor was end-capped with phenyl groups to reduce the trap density. These results showed that the trap density of N-type semiconducting polymers could be engineered to control the inherent charge-trapping capability and device performance for developing high-performance low-cost memory devices.

N-Type conjugated polymers based on naphthalene diimide (NDI) and various selenophene derivatives had been synthesized, characterized and evaluated as semiconductors for N-channel organic field-effect transistors (OFETs) by Ye-Jin Hwang.^51^ The new poly(naphthalene diimides) (PNDIs) have weight average molecular weights of 12–107 kDa with a polydispersity of 1.1–2.6. These PNDIs combined a constant electron affinity of 3.9 eV with an optical bandgap that varies from 1.7 eV in PNDISS (Fig. 2(b)) to 1.4 eV in ePNDIBS (Fig. 2(c)) due to intramolecular charge transfer. X-ray diffraction analysis of films of the polymers revealed a lamellar crystalline structure in which the lamellar interchain distance varied from 2.45 nm in PNDISS to 2.75 nm in PNDIBDS (Fig. 2(d)) while the p-stacking distance varied from 0.397 nm in PNDIBDS to 0.443 nm in PNDISS. Average field-effect electron mobility of 0.008 to 0.24 cm^2^ V^−1^ s^−1^ with high on/off current ratio (10^4^ to 10^6^) was observed from the bottom gate/top contact N-channel OFETs. A 3.4-fold enhancement in electron mobility was observed in phenyl end-capped and high molecular weight ePNDIBS whereas such end-capping hadn't largely effect on the electron mobility in PNDISS and PNDIBDS.

### 3.2 PDI-based N-type polymer semiconductor materials

**Fig. 3:**
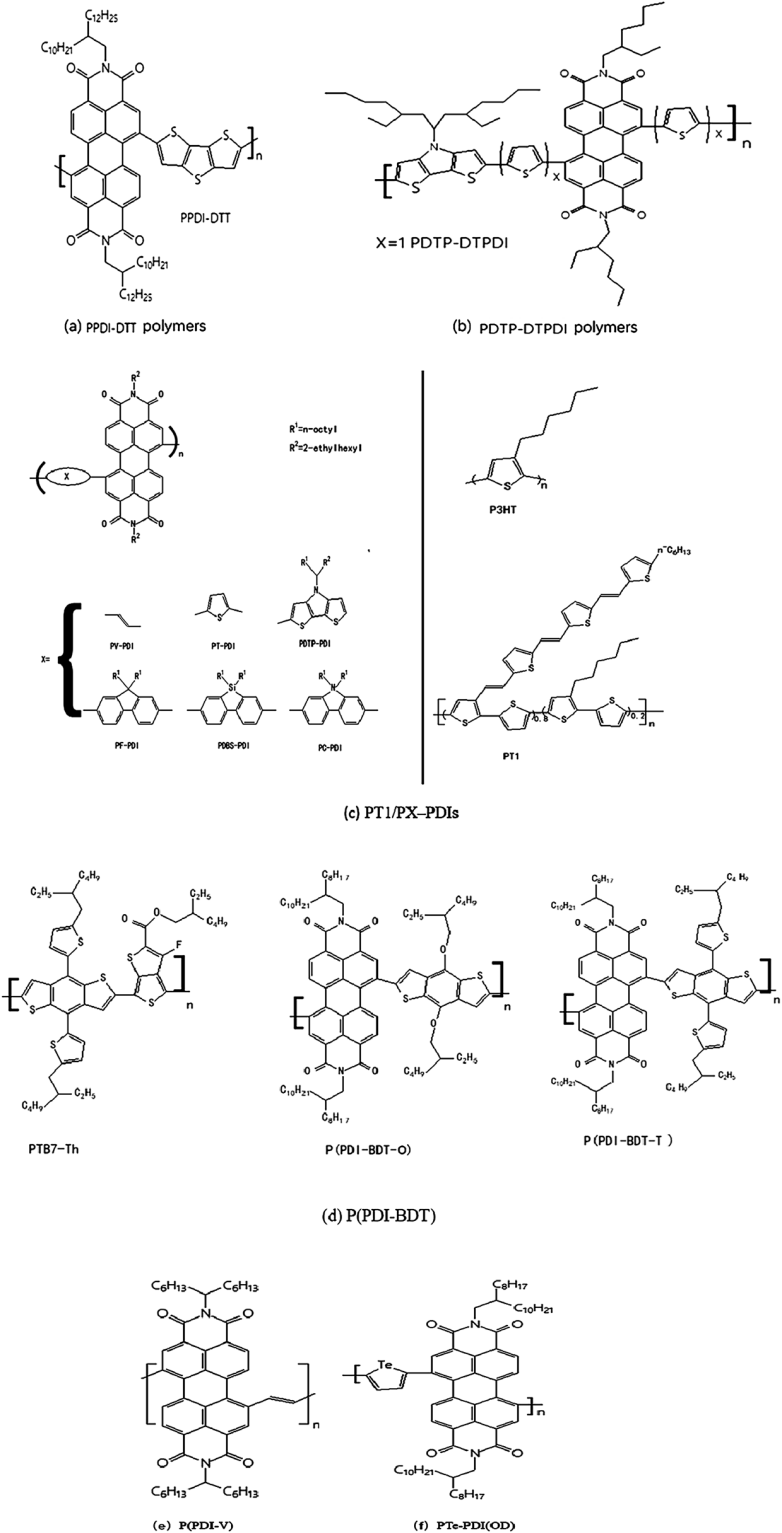
Representative molecular structures (a–f) of PDI-based N-type polymer semiconductors.

As early as 2007, Zhan *et al.*^52^ took the lead in constructing all-polymer solar cells using polymer PPDI-DTT based-perylene diimide (PDI) (Fig. 3(a)) as a receptor material. The corresponding energy conversion efficiency is 1%, and the electron mobility is up to 0.013 cm^2^ V^−1^ s^−1^.

In 2010, Zhou E *et al.*^53^ synthesized four types of perylene diimide-based electron acceptor materials. By changing the donor segment from fluorene to dithienopyrrole and/or introducing a thiophene unit as a spacer, the bandgap and energy levels of the resulting polymers could be tuned in a wide range. PDTP-DTPDI (Fig. 3(b)) exhibited the narrowest band gap of 1.24 eV, and the absorption edge extended to 1 mm. All-polymer solar cells based on these electron acceptors, blended with different electron donor polymers, namely, a polythiophene derivative (P_1_) and a low bandgap polymer (P_2_), were also investigated. P_1_: PDTP-PDI blends exhibited the highest power conversion efficiency of 0.93% under the illumination of AM 1.5 (100 mW cm^2^). The monochromatic photocurrent response of the photovoltaic device based on P_2_: PDTP-PDI blends extended to the near-infrared region up to 1 mm.

In 2011, Zhou, E *et al.*^54^ also investigated systematically all-PSCs based on six perylene diimide containing polymers (PX-PDIs) as acceptor materials and two polythiophene derivatives (P3HT and PT1) as donor materials. PT1/PX-PDIs (Fig. 3(c)) showed obvious improvement in their device performance compared to the corresponding P3HT/PX-PDI combinations because of the better film morphology of PT1/PX-PDIs and the lower HOMO energy level of PT1 than that of P3HT. Owing to the use of solvent mixtures and the thus obtained phase-separation control, the highest PCE of all-PSCs based on PT1/PX-PDI reached 2.23%, which was one of the best PCE values of polymer/polymer blend photovoltaic devices reported to date. Considering the large improvement in all-PSCs with the use of PT1 as donor material instead of P3HT, further investigation on the combination of P-type and N-type polymers could lead to a higher PCE, comparable to that of polymer/fullerene BHJs.

Controlling phase separation is one of the most critical issues in all-polymer solar cells as it limits the generation of free charge carriers and subsequently the device performance. Tuning processing parameters during device fabrication has been used to optimize phase separation. However, limited success has been achieved in this regard. In 2014, Zhou Y^55^ reported high performance all-polymer solar cells employing isoindigo-containing donor polymers and perylene tetracarboxlic di-imide (PTCDI)-containing acceptor polymers. Incorporation of polystyrene side chains into the donor polymer was found to assist in reducing the phase separation domain length scale. A direct correlation between the short circuit current (*J*_sc_) and the length scale of BHJ phase separation was observed. An average PCE of 4.2% from 20 devices with an average *J*_sc_ of 8.8 mA cm^−2^ was obtained. The highest PCE was 4.4%, with a *J*_sc_ as high as 9.0 mA cm^−2^, and *V*_oc_ of 1.04 V.

In 2015, a novel N-type two-dimension (2D)-conjugated polymer P(PDI-BDT-T) (Fig. 3(d)) based on bithienyl-benzodithiophene (BDT) and perylene diimide (PDI), was synthesized by Stille^56^ coupling reaction for the application as acceptor material in all-polymer solar cells (PSCs). P(PDI-BDT-T) exhibited broad absorption in the visible region with the optical bandgap (*E*_g_) of 1.64 eV, and a LUMO level of −3.89 eV which was similar with and slightly higher than that of PCBM, indicating that the polymer was suitable for the application as acceptor instead of PCBM in PSCs. The PSCs with P(PDI-BDT-T) as acceptor and PTB7-Th (Fig. 3(d)) as donor demonstrated a power conversion efficiency (PCE) of 4.71% with a *J*_sc_ of 11.51 mA cm^−2^, *V*_oc_ of 0.80 V, and FF of 51.1%. While the PCE of the PSCs based on the acceptor of a corresponding 1D-conjugated polymer P(PDI-BDT-O) (Fig. 3(d)) with alkoxy side chain on BDT unit was only 2.75% with a *J*_sc_ of 10.14 mA cm^−2^, *V*_oc_ of 0.72 V, and FF of 37.6%. These results indicated that the 2D-conjugated P(PDI-BDT-T) was a promising acceptor material for all polymer PSCs.

In 2016, Guo Y *et al.*^57^ designed and studied a new PDI polymer acceptor, that exhibits much-enhanced PCE in all-PSCs relative to the previously reported poly(PDI-thiophene). Main design rationale is to improve the planarity of the polymer backbone by reducing the steric hindrance near the bay region of PDI, which should increase the π–π stacking ability of the polymer backbone and thus enhancing electron transport. With such a design guideline, a new polymer, P(PDI-V) (Fig. 3(e)), composed of PDI units joined by vinylene linkers was synthesized. Solar cell devices were fabricated based on a well-known donor polymer named PTB7-Th and PDI-V, which achieved high efficiency of 7.57%.

Novel tellurophene-based N-type copolymers PTe-PDI(OD) (Fig. 3(f)), were synthesized by Lv, L.^49^ The copolymers demonstrated reversible interactions with bromine. Through tuning the building blocks and alkyl chains, together with device engineering, the maximum PCE of all-polymer solar cells improved from 2.8% to 4.3%, which was supported by photoluminescence, AFM, TEM, SCLC, and exciton dynamics studies. These results suggested that tellurophene-based N-type copolymers were promising electron acceptors for organic solar cells and potential sensor materials for bromine detection.

The monomer of PDI and NDI has some space steric hindrance, which causes the polymer skeleton structure to have a large twist angle. This is not conducive to the charge transfer. Moreover, the aggregation of the polymers which are prepared by PDI and NDI is usually serious, which poses a challenge to obtain good miscibility of acceptor materials in solar cell devices. Compared with amides, imides have the stronger electron-withdrawing ability and can reduce the energy level of polymers, which leads to possess unipolar properties. It is easy to design and synthesize monomer and also get N-type performance semiconductors.

### 3.3 BTI-based N-type polymer semiconductors

**Fig. 4:**
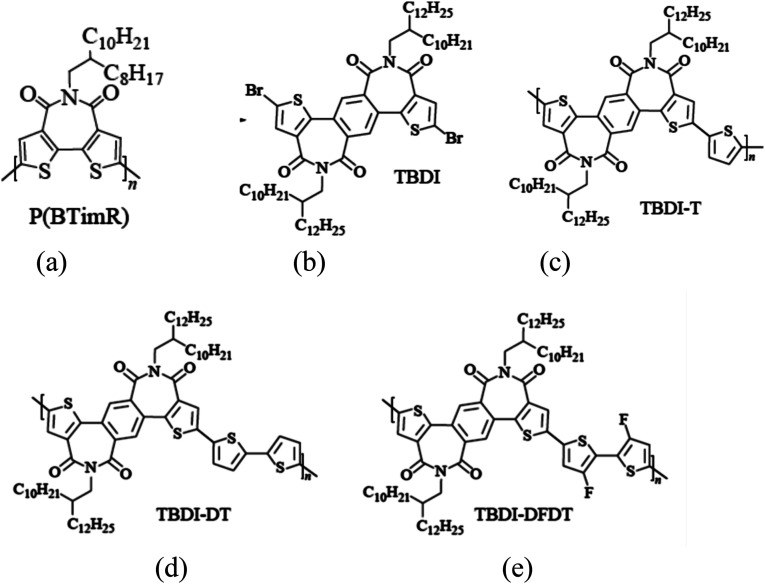
(a) P(BTimR), (b) BDI monomer and (c–e) BDI-based N-type polymer semiconductors.

In 2008, Marks team^23^ designed and synthesized bisthiophenoimide receptor unit (BTI). The imide was located between bisthiophene, which effectively reduced steric hindrance. At the same time, the BTI unit had good planarity and solubility, the corresponding homopolymer (PBTimR, Fig. 4(a)) obtained the electron mobility of 0.01 cm^2^ V^−1^ s^−1^ in transistors. The molecular weight of the polymer increased by changing the conditions of polymerization and post-treatment. The results showed that the crystallinity of the corresponding polymer film could be significantly improved by increasing the molecular weight. The electron mobility is 0.14 cm^2^ V^−1^ s^−1^.

Compared with PDI, NDI and amido building blocks, BTI dithiophenimide receptor building blocks not only effectively reduce the steric hindrance, have good planarity and tight molecular spacing, but also have strong electron pulling ability. Therefore, the design and synthesis of novel BTI-based polymers provide an excellent opportunity for the development of high-performance N-type semiconductors.

#### 3.3.1 Ladder polyimide-based N-type polymer semiconductor materials

Because of BTI's unique chemical structure, unique electronic properties and the superior device performances exhibited in its semiconductors, Chen J. H. *et al.*^58^ systematically worked on the design and development of BTI-based units, and designed and synthesized a series of trapezoidal semiconductor materials.

In 2017, a new dithiophenephthalimide (TBDI) was reported by Chen J. H. *et al.*^59^ Considering the electron-rich properties of thiophene, an electrically neutral benzene ring was introduced. The introduction of benzene rings could effectively reduce the orbital energy levels of the Frontier molecular, which was more conducive to the polymer to exhibit the N-type performances.

To understand the structure–performance relationship of TBDI-based polymer semiconductors more clearly, single crystals were obtained by solvent diffusion of short-chain TBDI (Fig. 4(b)). The corresponding copolymer semiconductors TBDI-T (Fig. 4(c)), TBDI-DT (Fig. 4(d)) and TBDI-DFDT (Fig. 4(e)) are obtained by the polymerization TBDI monomers with monothiophene, dithiophene and fluorinated dithiophene respectively. Due to the non-planar structure and intrinsic electronic properties of TBDI, the three copolymers show a wide optical bandgap and low HOMO energy level (<−5.5 eV). In terms of transistors, TBDI-DT shows bipolar performance, which may be due to the electric enrichment. With the increase of thiophene, the HOMO energy level of copolymer semiconductor is improved, which is more favorable for hole injection. However, the copolymer TBDI-T has less thiophene number, showing N-type transistor performance, and the electron mobility reaches 0.11 cm^2^ V^−1^ s^−1^. By introducing the fluorine atom of the electron donor unit, the TBDI-DFDT with double thiophene also shows a single N-type performance, with high electron mobility. The experimental results show that although the TBDI building block does not show a high coplanar skeleton structure, the corresponding copolymer semiconductors show good N-type properties due to the strong electron-withdrawing ability and tight molecular spacing of imides, which also proves that TBDI is a promising electron-deficient monomer and can be used to build high performance N-type wide-bandgap polymers semiconductor materials.

**Fig. 5:**
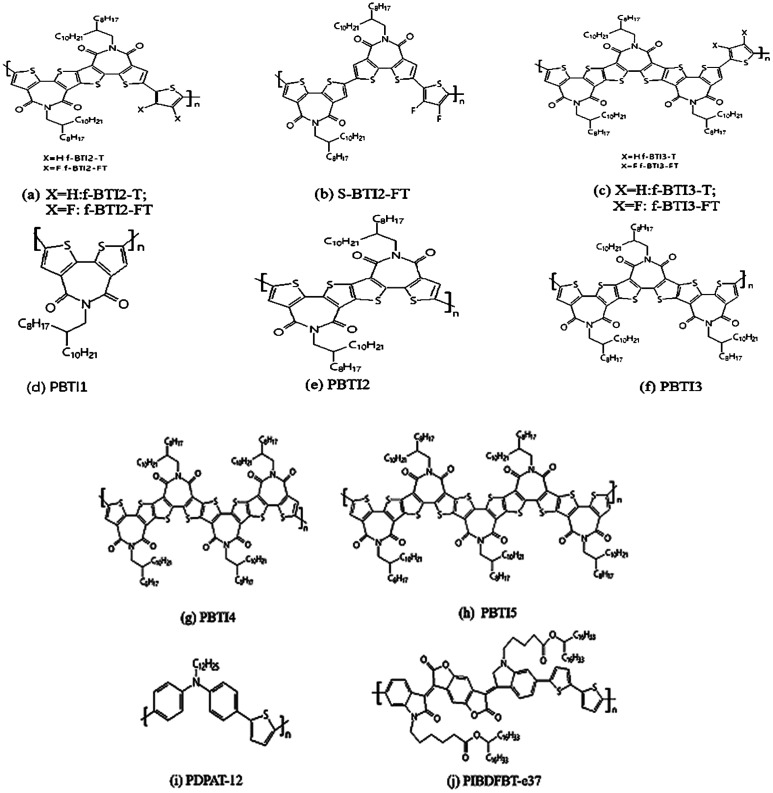
(a–c) Representative molecular structures of BTI-based N-type polymer semiconductors. (d–h) Chemical structures of polymer semiconductors with BTI1(or BTI)-BTI5, including both donor–acceptor (D–A) type copolymers, acceptor–acceptor (A–A) homopolymers and donor–donor (D–D) type copolymers. Structures of compounds (i) PDPAT-12 and (j) PIBDFBT-e37.

In 2017, Wang Y. F. *et al.*^60^ synthesized a series of fused ring BTI-based derivatives by utilizing the characteristics of easy modification and functionalization of the α-positions and β-positions of BTI monomers. The longest BTI5 contains 15 fused rings and 5 imide groups in fused-ring BTI-based derivatives. The schematic diagram of the structures of the BTI-based polymer is shown in Fig. 5.

Subsequently, Wang Y. F. *et al.*^30,60^ constructed high performance N-type polymer semiconductors based on fused ring BTI units as the core polymerized with different donor units, which were applied to organic field-effect transistors and all-polymer solar cells. The fused BTI2 acceptor unit was copolymerized with thiophene and bisfluorothiophene, respectively, they obtained the corresponding polymers f-BTI2-T and f-BTI2-FT (Fig. 5(a)), wherein f-BTI2-FT showed good N-type semiconductor properties. When it mixed with polymer donor PCE10, the corresponding all-polymer solar cell efficiency reached 6.85%. However, the cell efficiency of f-BTI2-T as an acceptor was only 4.34%. This was because the introduction to fluorine atoms into the electron-donating unit reduced the LUMO energy level of the polymer, and the bigger difference of LUMO energy level between the donor polymers and the acceptor polymers was more favorable for the separation of excitons, thereby improving the performance of solar cells.

Compared with the corresponding non-fused dimer polymer s-BTI2-FT (Fig. 5(b)), f-BTI2-FT showed obvious advantages in light absorption, mixed film morphology and electron mobility, which resulted in its device performance much better than s-BTI2-FT. This also showed the advantages of thick cyclization in the design of electronic-deficient units. Subsequently, the corresponding polymers f-BTI3-T and f-BTI3-FT^57^ (Fig. 5(c)) were obtained by the copolymerization of fused BTI3 acceptor units containing longer conjugation lengths with monothiophene and difluorothiophene, respectively. The introduction to fluorine atoms into the thiophene donor unit resulted in a blue shift of f-BTI3-FT absorption, the polymer had a wider optical bandgap and weakened the ability of absorption light. When PTB7-Th was used as the donor, f-BTI3-T and f-BTI3-FT were used as acceptors, and their solar cell efficiency PCE reached 8.98% and 7.33% respectively. This showed that, with the increase of the number in imides, not only reduced the front-line orbital energy levels (LUMO level) of the polymer, and improved the properties of N-type polymers, but also reduced the optical bandgap of the polymer and improved the light trapping ability. Besides, compared with the BTI2 unit with central symmetry, the BTI3 unit with axial symmetry made the corresponding polymer have more flexible polymer chain skeleton, which was more beneficial to the blend of the acceptor polymer and the donor polymer, and could improve the morphology of the mixed film and facilitate exciton separation and charge transfer. Moreover, the battery performance of f-BTI3-T as the receptor has already exceeded that of PC71BM, which mainly benefits the increase in *V*_oc_ (1.03 V). It predicts the great potential of the fused-ring BTI-based N-type polymer as acceptor material in all-polymer solar cells.

Based on the above strategy, Wang Y. F. *et al.*^26,61^ had reported a series of BTIn-based all-acceptor homopolymers (PBTI_*n*_, *n* = 1–5) (Fig. 5d–h). Compared with the previously reported results, transistor performance had greatly improved, and the highest electron mobility of PBTI1 was 3.71 cm^2^ V^−1^ s^−1^. In addition to the optimization of the preparation condition of the device, the Stille coupling reaction was used to replace the Yamamoto reaction, and the molecular weight of the corresponding polymer was nearly doubled. Besides, the Stille coupling reaction reduced the Br atom content at the end of the corresponding polymer, thus reduced the negative influence of Br atom on the properties of Polymer semiconductors, too. In 2019, Chen W. *et al.*^62^ applied PBTI as a grain boundary passivation in perovskite solar cells. PBTI improved the crystalline properties of the films and effectively reduced the defects and the carrier recombination, thereby improving PCE and greatly raising the light stability of the device.

He Y. H. *et al.*^63^ synthesized an aromatic amine-containing polymer PDPAT-12 (Fig. 5(i)) and its use as an effective additive to an ambipolar polymer semiconductor PIBDFBT-e37 (Fig. 5(j)) to achieve unipolar N-type charge transport in organic thin-film transistors (OTFTs). By adding 10 wt% PDPAT-12 to PIBDFBT-e37, the OTFTs achieved unipolar N-type charge transport with electron mobility of up to 0.42 cm^2^ V^−1^ s^−1^ in nitrogen and 0.19 cm^2^ V^−1^ s^−1^ in air. Besides, the stability of the polymer blend in ambient air was improved (retaining 50% of its initial electron mobility after exposed in air for 28 days) than the pristine PIBDFBT-e37 (retaining 13% of its initial electron mobility under the same conditions) and the PIBDFBT-e37: PEI (polyethylenimine) blend, which lost transistor performance as soon as it is exposed to ambient air. The results showed that in the OTFTs, the use of the aromatic amine-containing polymer as an additive can convert an ambipolar polymer into an N-type unipolar polymer, which greatly improves air stability.

#### 3.3.2 β-Fluorine substituted dithiophenoimide N-type polymer semiconductor materials

**Fig. 6:**
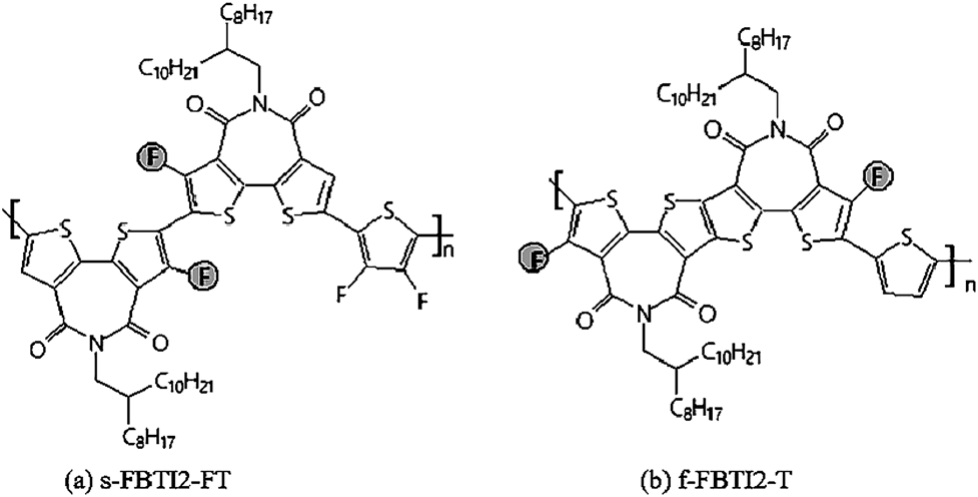
Chemical structures of (a) s-FBTI2-FT and (b) f-FBTI2-T.

Recently, a new electron-deficient building block s-FBTI2 had been constructed by introducing a fluorine atom into a thiophenimide dimer.^64^ The introduction of a fluorine atom contributed to enhancing the non-covalent bond of S⋯F in the molecule, thereby increased the molecular backbone coplanarity. Then s-FBTI2 was polymerized with difluorothiophene to afford polymer s-FBTI2-FT (Fig. 6(a)). The polymer s-FBTI2-FT had a lower LUMO energy level than the fluorine-free dimer-based polymer, which fully demonstrated that the introduction of fluorine atoms on the imide could make the whole molecular skeleton have a stronger electron-withdrawing ability. When the polymer s-FBTI2-FT was applied to a transistor, it showed good electron mobility with 2.73 cm^2^ V^−1^ s^−1^. The *I*_offs_ of the s-FBTI2-FT transistor were small (10^−9^ to 10^−10^ A), and the *I*_on_/*I*_offs_ (10^6^ to 10^7^) were very high. This performance was much higher than that of the thiophene dimer polymer s-BTI2-FT without fluorine.^60^ s-FBTI2-FT was used as an acceptor material, which had good energy conversion efficiency (PCE = 6.50%) in all-polymer solar cells, and had a high open voltage (*V*_oc_ = 1.04 V), energy loss was only 0.54 eV. It was worth noting that the energy conversion efficiency of such a high-polymer battery was obtained by not using any additives and not need annealing conditions, which not only simplified the device preparation process but also was more suitable for application in large-area flexible devices.

In 2019, Sun H. L. *et al.*^65^ synthesized a new fluorine-containing fused cyclic imide f-FBTI2-T. The introduction of fluorine atoms and bromine atoms on the electron-deficient unit was very challenging due to low chemical reactivity and certain steric hindrance effects. This new building block was introduced into the polymer backbone to get the polymer f-FBTI2-T (Fig. 6(b)). Compared to polymers BTI without F atoms, f-FBTI2-T had a low LUMO energy level and a narrow optical bandgap. It was used as an acceptor material in all-polymer solar cells with high energy conversion efficiency (PCE = 8.1%), large open-circuit voltage (*V*_oc_ = 1.05 V) and low energy loss (only 0.53 eV). This was the smallest energy loss reported in the all-polymer solar cells with above 8% PCE, which indicated that fluorinated imide was a very effective method for designing novel electron-deficient building blocks.

**Fig. 7:**
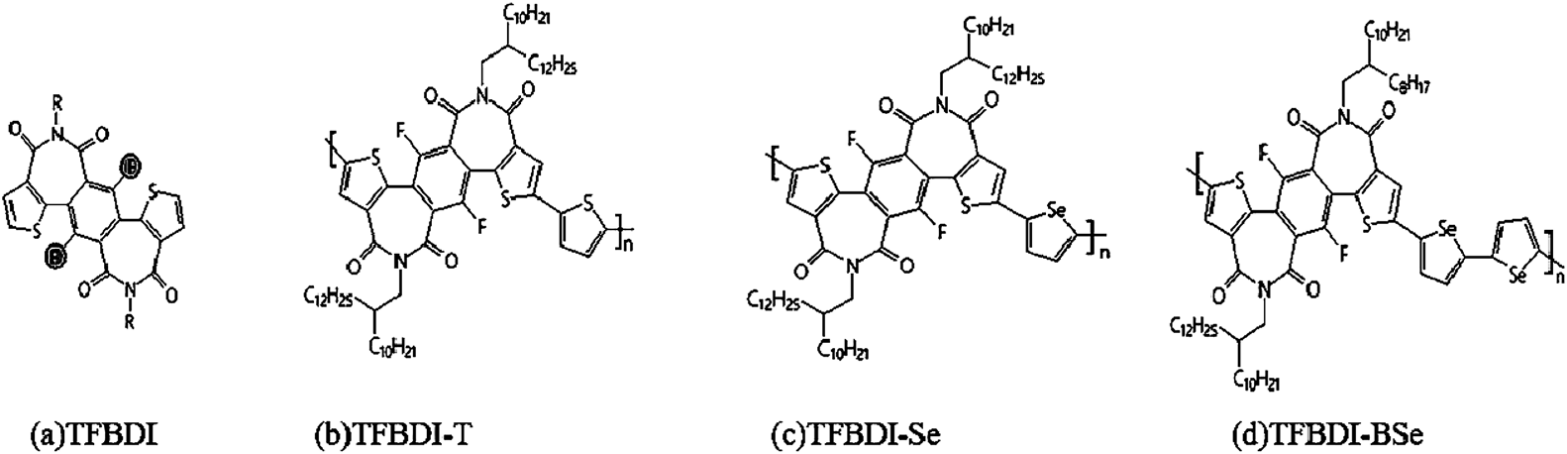
Some monomers of fluorinated dithienylbenzodiimides (a–d).

Imide functionalization is one of the most effective approaches to develop electron-deficient building blocks for constructing N-type organic semiconductors. Driven by the appealing properties of imide-functionalized dithienylbenzodiimide (TBDI) and the promising device performance of TBDI-based polymers, a novel acceptor with increased electron affinity, fluorinated dithienylbenzodiimide (TFBDI) (Fig. 7(a)), was designed with the hydrogen replaced by fluorine on the benzene core, and the synthetic challenges associated with this highly electron-deficient fluorinated imide building block are successfully overcome.^66^ TFBDI showed suppressed Frontier molecular orbital (FMO) energy levels as compared with TBDI. Copolymerizing this new electron-withdrawing TBDI with various donor co-units afforded a series of N-type polymer semiconductors: TFBDI-T (Fig. 7(b)), TFBDI-Se (Fig. 7(c)), and TFBDI-BSe (Fig. 7(d)). All these polymers containing TFBDI exhibited a lower-lying lowest unoccupied molecular orbital (LUMO) energy level than the polymer analogue without fluorine. When applied in organic thin-film transistors (OTFTs), three polymers showed unipolar electron transport with large on-current/off-current ratios (*I*_on_/*I*_offs_) of 10^5^ to 10^7^. Among them, the selenophene-based polymer TFBDI-Se with the deepest-positioned LUMO and optimal chain stacking exhibited the highest electron mobility of 0.30 cm^2^ V^−1^ s^−1^. This result demonstrates that the new TFBDI is a highly appealing electron-deficient unit for enabling N-type polymer semiconductors and the fluorination of imide-functionalized arenes offers an effective approach to develop more electron-deficient building blocks in organic electronics.

### 3.4 Thiazolimide-based N-type polymer semiconductor materials

Compared with electron-rich thiophene, thiazole is more electron-deficient and can effectively reduce the steric hindrance. Shi Y. Q. *et al.*^25^ succeeded in replacing thiazoles with thiophenes in thiophenimide dimer units. Because of the formation of the intramolecular non-covalent S⋯N bond by introducing thiazole, which makes the thiazole imide unit DTzTI further improve the planarity of the molecule, meanwhile further reduce the energy level, thereby contributing to device performance. Single crystal analysis showed that the monomer had good planarity, and the non-covalent bond of S⋯N was beneficial to improve the coplanarity of polymer skeleton, which enhances the crystallinity of the polymer material and promotes the charge transfer. Because of its small steric resistance, DTzTI was suitable for the construction of A–A type polymer semiconductors, and the obtained polymer PDTzTI also showed good solubility and moderate molecular weight (7.0 kDa). Electrochemical tests revealed that PDTzTI had low HOMO (−5.78 eV) level and low LUMO (−3.77 eV) level, and the low HOMO could effectively suppress hole injection. Therefore, OFET devices prepared by this polymer had high electron mobility (1.61 cm^2^ V^−1^ s^−1^), low leakage current *I*_offs_ (10^−10^ to 10^−11^ A) and large switching ratio *I*_on_/*I*_offs_ (10^7^ to 10^8^).

**Fig. 8:**
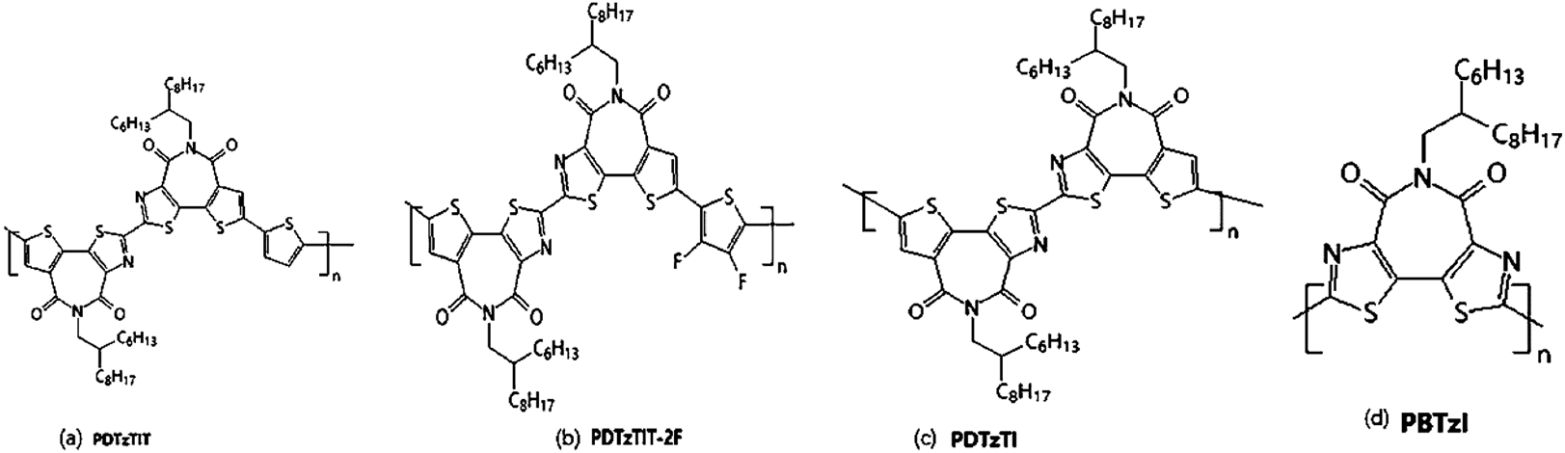
Chemical structures of (a) PDTzTIT, (b) PDTzTIT-2F, (c) PDTzTI and (d) PBTzI.

Compared to the polymers with A–A structures, high-performance N-type polymers based on D–A structures generally have high HOMO levels, making polymers susceptible to oxidative doping, which resulted in transistor devices having lower switching ratios (*I*_on_/*I*_offs_ < 10^5^) and high leakage current (*I*_offs_ > 10^−8^ A). Therefore, the study showed that the thiazolimide polymer semiconductor with A–A structure was conducive to the improvement of electronic transmission performance, while ensuring high switch ratio and low leakage current. And then DTzTI was used to polymerize with monothiophene and difluorothiophene respectively to obtain the corresponding D–A–A type polymer PDTzTIT (Fig. 8(a)) and PDTzTIT-2F (Fig. 8(b)). The electron mobility of the two transistors was 0.20 and 0.91 cm^2^ V^−1^ s^−1^, respectively. The LUMO level of these D–A–A polymer semiconductors was significantly higher than that of the A–A structure, and the transistor performance was lower than that of PDTzTI (Fig. 8(c)). This also fully demonstrated that, with the increase of the electron-deficient unit in the polymer backbone, the N-type properties of the polymer increase gradually. Subsequently, a more electron-deficient bithiazole imide BTzI^28^ was synthesized from 2-bromothiazole as the raw material. The total receptor homopolymer was constructed by using BTzI monomer (Fig. 8(d)). By introducing nitrogen atom, the LUMO and HOMO energy levels of PBTzI could be effectively reduced. The LUMO/HOMO energy levels of PBTzI could reach −3.94/−6.17 eV. This low energy level could effectively inhibit the hole injection and make PBTzI had very low leakage current *I*_offs_ (10^−10^ to 10^−11^ A). However, the mobility of PBTzI was only 0.015 cm^2^ V^−1^ s^−1^. It may be due to the lower reaction activity of the second site of thiazole ring and the polymer with the smaller molecular weight was obtained.

**Fig. 9:**
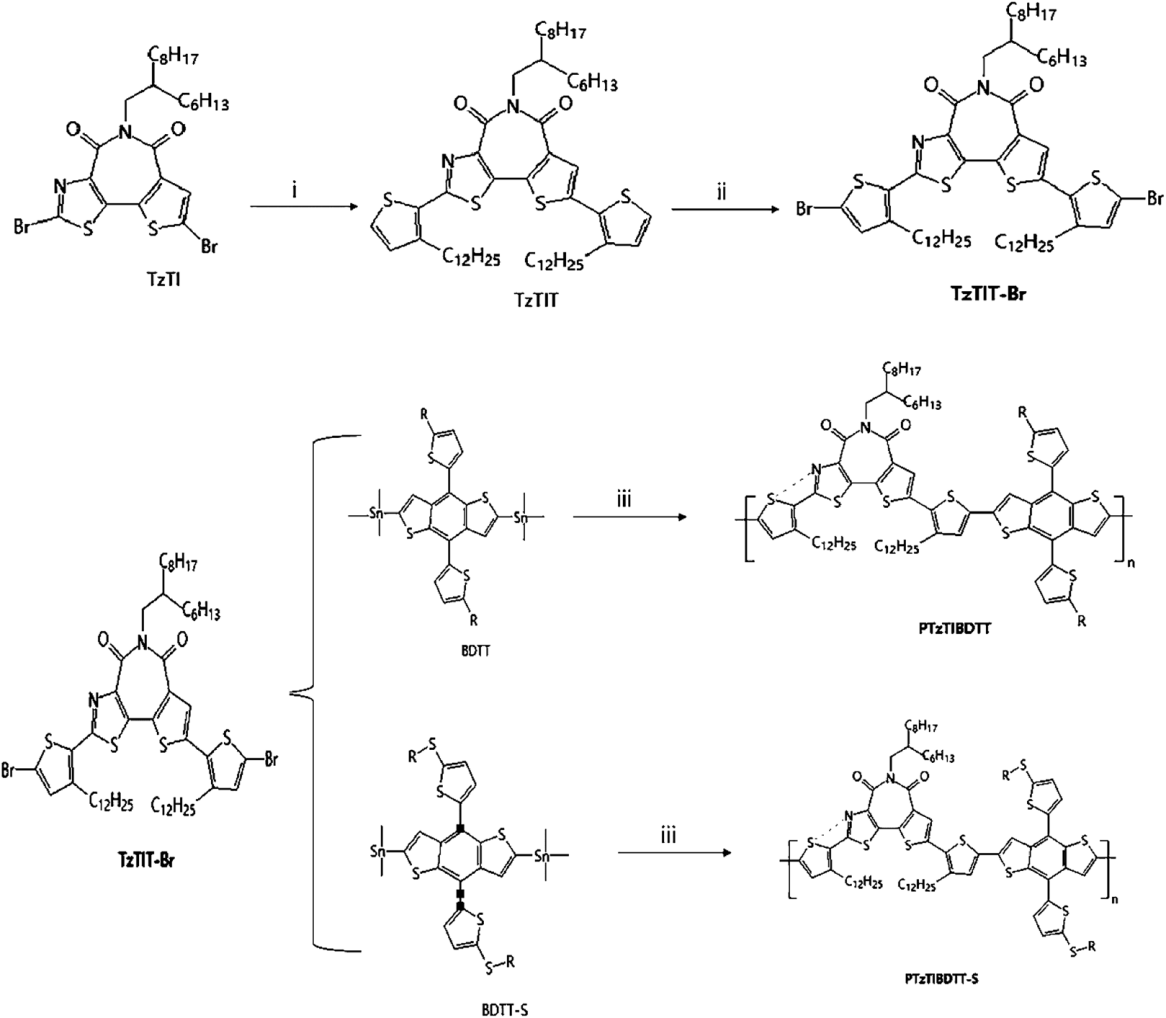
Synthetic route to the thiazolothienyl imide based polymers PTzTIBDTT and PTzTIBDTT-S.

Two new wide-bandgap copolymers PTzTIBDTT and PTzTIBDTT-S (Fig. 9) based on thiazolothienyl imide (TzTI) and benzodithiophene with different side chains were synthesized and characterized for polymer solar cells (PSCs) by Shi Y. Q., *et al.*^67^ Besides, the incorporation of TzTI can trigger noncovalent N⋯S interactions in the molecule to produce the self-flattened polymer backbone, which should be beneficial to the realization of ordered molecular stacking and effective charge transport. Because of its powerful electron-withdrawing effect, the participation of the TzTI unit significantly decreased the polymer HOMO levels to −5.65 and −5.69 eV for PTzTIBDTT and PTzTIBDTT-S, respectively. The PSCs containing the PTzTIBDTT:PC71BM active layer showed 8.00% power conversion efficiency (PCE) and 0.90 V *V*_oc_. As far as they were known, the PCE was one of the highest values of fullerene PSCs based on imide-containing polymer donors. This work not only proved that thiazolothienyl imide was an excellent building block for creating high-performance wide-bandgap photovoltaic polymer semiconductors but also showed that a noncovalent N⋯S conformational lock was an effective molecular design method for enabling polymer semiconductors with a planar backbone for highly efficient PSCs.

## 4 Summary and prospect

After decades of development, N-type polymer semiconductors have made great progress in the diversity of molecular structure, which significantly improves the performance of organic field-effect transistors and all polymer solar cells. But compared with P-type polymer semiconductor, there is still a big gap. At present, the methods to realize N-type properties of polymer semiconductor materials are as follows: (1) designing a new type of electron deficient unit; (2) introducing strong electron withdrawing groups, such as: –F, –CN, =O, and so on; (3) developing polymer semiconductors with full acceptor backbone structure; (4) improving the symmetry of side chain and conjugated backbone.^68–70^

Recently, N-type polymer P (NDI2DT-TTCN) has achieved the highest energy conversion efficiency (PCE = 17%) as an electron transport layer.^71^ The backbone of P (NDI2DT-TTCN) polymer contains NDI unit and thiophene dicyano unit. The LUMO level is as low as −4.14 eV, which is the lowest level in the electron transport layer. In 2020, Shi *et al.*^72^ recently synthesized a diarylated electron deficient bisthiophene imide (BTI-BTI2) monomer, and obtained homopolymer PBTI and copolymer P (BTI-BTI2) by static coupling with acylated co unit, both of which have high molecular weight receptor main chain. Due to the improvement of electronic properties and the increase of molecular weight, these two polymers exhibit excellent unipolar N-type characteristics in transistors with electron mobility up to 2.60 cm^2^ V^−1^ s^−1^. When PBTI and P (BTI-BTI2) were used as acceptors in all polymer solar cells, high power conversion efficiencies of 6.67% and 8.61% were obtained, respectively.

Besides, the stability of N-type polymer devices is obviously insufficient, so it is necessary to combine the development of new materials and device process to improve its stability. Therefore, the design and development of high performance N-type polymer semiconductors is still an important topic in the field of organic electronics. Imide-functionalized acceptor–acceptor copolymers as efficient electron transport layers was used in high-performance perovskite solar cells.^73^ This work provides important guidelines for designing N-type polymers to replace the conventional [6,6]-phenyl-C61-butyric acid methyl ester as efficient electron transport layers for high-performance perovskite solar cells with improved stability. The research indicate N-type polymer semiconductors has been extended to the field of perovskite solar cells.

## Conflicts of interest

There are no conflicts to declare.

## Supplementary Material

## References

[cit1] Chiang C. K., Fincher C. R., Park Y. W., Heeger A. J., Shirakawa H., Louis E. J., Gau S. C., MacDiarmid A. G. (1978). Electrical Conductivity in Doped Polyacetylene. Phys. Rev. Lett..

[cit2] Ando T., Koezuka H., Tsumura A. (1986). Macromolecular electronic device: field-effect transistor with a polythiophene thin film. Appl. Phys. Lett..

[cit3] Tang C. W. (1986). Two-layer organic photovoltaic cell. Appl. Phys. Lett..

[cit4] Chen J., Cao Y. (2009). Acc Chem Res, Development of Novel Conjugated Donor Polymers for High-Efficiency Bulk-Heterojunction Photovoltaic Devices. ACS Publications.

[cit5] Li Y. (2012). Molecular design of photovoltaic materials for polymer solar cells: toward suitable electronic energy levels and broad absorption. Acc. Chem. Res..

[cit6] Cheng P., Zhan X. (2016). Stability of organic solar cells: challenges and strategies. Chem. Soc. Rev..

[cit7] Lin Y., Li Y., Zhan X. (2012). Small molecule semiconductors for high-efficiency organic photovoltaics. Chem. Soc. Rev..

[cit8] Zhan X., Zhu D. (2010). Conjugated polymers for high-efficiency organic photovoltaics. Polym. Chem..

[cit9] Zhao X., Zhan X. (2011). Electron transporting semiconducting polymers in organic electronics. Chem. Soc. Rev..

[cit10] Chen Y., Wan X., Long G. (2013). High performance photovoltaic applications using solution-processed small molecules. Acc. Chem. Res..

[cit11] Lin Y., Zhan X. (2016). Oligomer Molecules for Efficient Organic Photovoltaics. Acc. Chem. Res..

[cit12] Ye L., Zhang S., Huo L., Zhang M., Hou J. (2014). Molecular design toward highly efficient photovoltaic polymers based on two-dimensional conjugated benzodithiophene. Acc. Chem. Res..

[cit13] Cheng P., Zhan X. (2015). Versatile third components for efficient and stable organic solar cells. Mater. Horiz..

[cit14] Chen H. J., Guo Y. L., Yu G., Zhao Y., Zhang J., Gao D., Liu H. T., Liu Y. Q. (2012). Highly π-extended copolymers with diketopyrrolopyrrole moieties for high-performance field-effect transistors. Adv. Mater..

[cit15] Li J., Zhao Y., Tan H. S., Guo Y. L., Di C. A., Yu G., Liu Y. Q., Lin M., Lim S. H., Zhou Y. H., Su H. B., Ong B. S. (2012). A stable solution-processed polymer semiconductor with record high-mobility for printed transistors. Sci. Rep..

[cit16] Mei J. G., Diao Y., Appleton A. L., Fang L., Bao Z. N. (2013). Integrated materials design of organic semiconductors for field-effect transistors. J. Am. Chem. Soc..

[cit17] Yang J., Zhao Z. Y., Wang S., Guo Y. L., Liu Y. Q. (2018). Insight into High Performance Conjugated Polymers for Organic Field-Effect Transistors. Chem.

[cit18] Xu X. M., Yao Y. F., Shan B. W., Gu X., Liu D. Q., Liu J. Y., Xu J. B., Zhao N., Hu W. P., Miao Q. (2016). Electron Mobility Exceeding 10cm2V-1 s-1 and Band-Like Charge Transport in Solution-Processed n-Channel Organic Thin-Film Transistors. Adv. Mater..

[cit19] Zhao Y., Guo Y. L., Liu Y. Q. (2013). 25th anniversary article: recent advances in N-type and ambipolar organic field-effect transistors. Adv. Mater..

[cit20] Zaumseil J., Sirringhaus H. (2007). Electron and ambipolar transport in organic field-effect transistors. Chem. Rev..

[cit21] Yang J., Chen J., Sun Y., Shi L., Guo Y., Wang S., Liu Y. (2017). Isoindigo-Based Polymers with Small Effective Masses for High-Mobility Ambipolar Field-Effect Transistors. Acta Polym. Sin..

[cit22] Guo X. G., Watson M. D. (2008). Conjugated polymers from naphthalene bisimide. Org. Lett..

[cit23] Letizia J. A., Salata M. R., Tribout C. M., Facchetti A., Ratner M. A., Marks T. J. (2008). n-channel polymers by design: optimizing the interplay of solubilizing substituents, crystal packing, and field-effect transistor characteristics in polymeric bithiophene-imide semiconductors. J. Am. Chem. Soc..

[cit24] Guo X. G., Ortiz R. P., Zheng Y., Hu Y., Noh Y. Y., Baeg K. J., Facchetti A., Marks T. J. (2011). Bithiophene-imide-based polymeric semiconductors for field-effect transistors: synthesis, structure-property correlations, charge carrier polarity, and device stability. J. Am. Chem. Soc..

[cit25] Zhou N. J., Guo X. G., Ortiz R. P., Harschneck T., Manley E. F., Lou S. J., Hartnett P. E., Yu X. G., Horwitz N. E., Burrezo P. M., Aldrich T. J., López Navarrete J. T., Wasielewski M. R., Chen L. X., Chang R. P. H., Facchetti A. (2015). Marked consequences of systematic oligothiophene catenation in thieno[3,4-c]pyrrole-4,6-dione and bithiopheneimide photovoltaic copolymers. J. Am. Chem. Soc..

[cit26] Wang Y. F., Guo H., Harbuzaru A., Uddin M. A., Arrechea-Marcos I., Ling S. H., Yu J. W., Tang Y. M., Sun H. L., López Navarrete J. T., Ortiz R. P., Woo H. Y., Guo X. G. (2018). (Semi)ladder-Type Bithiophene Imide-Based All-Acceptor Semiconductors: Synthesis, Structure-Property Correlations, and Unipolar N-type Transistor Performance. J. Am. Chem. Soc..

[cit27] Shi Y. Q., Guo H., Qin M. C., Zhao J. Y., Wang Y. X., Wang H., Wang Y. L., Facchetti A., Lu X. H., Guo X. G. (2018). Thiazole Imide-Based All-Acceptor Homopolymer: Achieving High-Performance Unipolar Electron Transport in Organic Thin-Film Transistor. Adv. Mater..

[cit28] Shi Y. Q., Guo H., Qin M. C., Wang Y. X., Zhao J. Y., Sun H. L., Wang H., Wang Y. L., Zhou X., Facchetti A., Lu X. H., Zhou M., Guo X. G. (2018). Imide-Functionalized Thiazole-Based Polymer Semiconductors: Synthesis, Structure–Property Correlations, Charge Carrier Polarity, and Thin-Film Transistor Performance. Chem. Mater..

[cit29] Saito M., Osaka I., Suda Y., Yoshida H., Takimiya K. (2016). Dithienylthienothiophenebisimide, a Versatile Electron-Deficient Unit for Semiconducting Polymers. Adv. Mater..

[cit30] Wang Y. F., Guo H., Ling S. H., Arrechea-Marcos I., Wang Y. X., López Navarrete J. T., Ortiz R. P., Guo X. G. (2017). Ladder-type Heteroarenes: Up to 15 Rings with Five Imide Groups. Angew. Chem., Int. Ed..

[cit31] Guo X. G., Watson M. D. (2011). Pyromellitic
Diimide-Based Donor-Acceptor Poly(phenylene ethynylene)s. Macromolecules.

[cit32] Guo X. G., Kim F. S., Seger M. J., Jenekhe S. A., Watson M. D. (2012). Design, Naphthalene Diimide-Based Polymer Semiconductors: Synthesis, Structure–Property Correlations, and n-Channel and Ambipolar Field-Effect Transistors. Chem. Mater..

[cit33] Dou C. D., Long X. J., Ding Z. C., Xie Z. Y., Liu J., Wang L. X. (2016). Morphology of small molecular donor/polymer acceptor blends in organic solar cells: effect of the π–π stacking capability of the small molecular donors. Angew. Chem., Int. Ed..

[cit34] Long X. J., Gao Y., Tian H. K., Dou C. D., Yan D. H., Geng Y. H., Liu J., Wang L. X. (2017). Electron-transporting polymers based on a double B←N bridged bipyridine (BNBP) unit. Chem. Commun..

[cit35] Yu J. W., Ornelas J. L., Tang Y. M., Uddin M. A., Guo H., Yu S. M., Wang Y. L., Woo H. Y., Zhang S. M., Xing G. C., Guo X. G., Huang W. (2017). 2,1,3-Benzothiadiazole-5,6-dicarboxylicimide-Based Polymer Semiconductors for Organic Thin-Film Transistors and Polymer Solar Cells. ACS Appl. Mater. Interfaces.

[cit36] Osaka I., Shimawaki M., Mori H., Doi I., Miyazaki E., Koganezawa T., Takimiya K. (2012). Synthesis, characterization, and transistor and solar cell applications of a naphthobisthiadiazole-based semiconducting polymer. J. Am. Chem. Soc..

[cit37] Zhang Q. Q., Kelly M. A., Bauer N., You W. (2017). The impact of fluorination on both donor polymer and non-fullerene acceptor: The more fluorine, the merrier. Acc. Chem. Res..

[cit38] Liu J., Ye G., van der Zee B., Dong J. J., Qiu X. K., Liu Y. R., Portale G., Chiechi R. C., Koster L. J. A. (2018). Perovskite Solar Cells: A Cryogenic Process for Antisolvent-Free High-Performance Perovskite Solar Cells. Adv. Mater..

[cit39] Liao Q. G., Wang Y. L., Uddin M. A., Chen J. H., Guo H., Shi S. B., Wang Y., Woo H. Y., Guo X. G. (2018). Drastic Effects of Fluorination on Backbone Conformation of Head-to-Head Bithiophene-Based Polymer Semiconductors. ACS Macro Lett..

[cit40] Huang H., Yang L., Facchetti A., Marks T. J. (2017). Organic and polymeric semiconductors enhanced by noncovalent conformational locks. Chem. Rev..

[cit41] Yan H., Chen Z. H., Zheng Y., Newman C., Quinn J. R., Dötz F., Kastler M., Facchetti A. (2009). A high-mobility electron-transporting polymer for printed transistors. Nature.

[cit42] Kim F. S., Guo X. G., Watson M. D., Jenekhe S. A. (2010). Organic Electronics: High-mobility Ambipolar Transistors and High-gain Inverters from a Donor–Acceptor Copolymer Semiconductor. Adv. Mater..

[cit43] Fukutomi Y., Nakano M., Hu J. Y., Osaka I., Takimiya K. (2013). Naphthodithiophenediimide (NDTI): synthesis, structure, and applications. J. Am. Chem. Soc..

[cit44] Kang B., Kim R., Lee S. B., Kwon S., Kang K., Kim Y. H., Cho K. (2016). Side-chain-induced rigid backbone organization of polymer semiconductors through semifluoroalkyl sde chains. J. Am. Chem. Soc..

[cit45] Wang Y., Hasegawa T., Matsumoto H., Michinobu T. (2019). Significant Improvement of Unipolar N-type Transistor Performances by Manipulating the Coplanar Backbone Conformation of Electron-Deficient Polymers *via* Hydrogen Bonding. J. Am. Chem. Soc..

[cit46] Di Pietro R., Fazzi D., Kehoe T. B. (2012). Spectroscopic Investigation of Oxygen- and Water-Induced Electron Trapping and Charge Transport Instabilities in N-type Polymer Semiconductors. J. Am. Chem. Soc..

[cit47] Kim Yu J. (2018). A control of structural morphology *via* introducing insulating polymers in N-type P(NDI2OD-T2) semiconductor. J. Mater. Sci..

[cit48] Selvam S., Earmme T., Murari N. M., Jenekhe S. A. (2014). Naphthobisthiazole diimide-based N-type polymer semiconductors: synthesis, p-stacking, field-effect charge transport, and all-polymer solar cells. Polym. Chem..

[cit49] Lv L., Wang X., Wang X., Yang L., Dong T., Yang Z., Huang H. (2016). Tellurophene-Based N-type Copolymers for Photovoltaic Applications. ACS Appl. Mater. Interfaces.

[cit50] Murari N. M., Hwang Y.-J., Kim F. S., Jenekhe S. A. (2016). Organic nonvolatile memory devices utilizing intrinsic chargetrapping phenomena in an N-type polymer semiconductor. Org. Electron..

[cit51] Hwang Ye-J., Murari N. M., Jenekhe S. A. (2013). New N-type polymer semiconductors based on naphthalene diimide and selenophene derivatives for organic field-effect transistors. Polym. Chem..

[cit52] Zhan X. W., Tan Z. A., Domercq B., An Z. S., Zhang X., Barlow S., Li Y. F., Zhu D. B., Kippelen B., Marder S. R. (2007). Small molecular non-fullerene electron acceptors for P3HT-based bulk-heterojunction solar cells. J. Am. Chem. Soc..

[cit53] Zhou E., Tajima K., Yang C., Hashimoto K. (2010). Band Gap and Molecular Energy Level Control of Perylene Diimide-based Donor–Acceptor Copolymers for All-Polymer Solar Cells. J. Mater. Chem..

[cit54] Zhou E., Cong J., Wei Q., Tajima K., Yang C., Hashimoto K. (2011). All-Polymer Solar Cells from Perylene Diimide Based Copolymers: Material Design and Phase Separation Control. Angew. Chem., Int. Ed..

[cit55] Zhou Y., Kurosawa T., Ma W., Guo Y., Fang L., Vandewal K., Diao Y., Wang C., Yan Q., Reinspach J., Mei J., Appleton A. L., Koleilat G. I., Gao Y., Mannsfeld S. C., Salleo A., Ade H., Zhao D., Bao Z. (2014). High Performance All-Polymer Solar Cell *via* Polymer Side-chain Engineering. Adv. Mater..

[cit56] Zhang Y., Wan Q., Guo X., Li W., Guo B., Zhang M., Li Y. (2015). Synthesis and Photovoltaic. Properties of An N-type Two-dimension-conjugated Polymer based on Perylene Diimide and Benzodithiophene with Thiophene Conjugated Side Chains. J. Mater. Chem. A.

[cit57] Guo Y., Li Y., Awartani O., Zhao J., Han H., Ade H., Zhao D., Yan H. (2016). A Vinylene-Bridged Perylenediimide-Based Polymeric Acceptor Enabling Efficient All-Polymer Solar Cells Processed under Ambient Conditions. Adv. Mater..

[cit58] Chen J. H., Yang K., Zhou X., Guo X. G. (2018). Recent Advances in Laddertype Heteroarene-based Organic. Chem.–Asian J..

[cit59] Chen J. H., Zhang X. H., Wang G., Uddin M. A., Tang Y. M., Wang Y. L., Liao Q. G., Facchetti A., Marks T. J., Guo X. G. (2017). Dithienylbenzodiimide: a new electron-deficient unit for N-type polymer semiconductors. J. Mater. Chem. C.

[cit60] Wang Y. F., Yan Z. L., Guo H., Uddin M. A., Ling S. H., Zhou X., Su H. M., Dai J. F., Woo H. Y., Guo X. G. (2017). Effects of Bithiophene Imide Fusion on the Device Performance of Organic Thin-Film Transistors and All-Polymer Solar Cells. Angew. Chem., Int. Ed..

[cit61] Wang Y. F., Yan Z. L., Uddin M. A., Zhou X., Yang K., Tang Y. M., Liu B., Shi Y. Q., Sun H. L., Deng A. Y., Dai J. F., Woo H. Y., Guo X. G. (2019). Triimide-Functionalized n-Type Polymer Semiconductors Enabling All-Polymer Solar Cells with Power Conversion Efficiencies Approaching 9%. Sol. RRL.

[cit62] Chen W., Wang Y. F., Pang G. T., Koh C. W., Djurišić A. B., Wu Y. H., Tu B., Liu F. Z., Chen R., Woo H. Y., Guo X. G., He Z. B. (2019). Adv. Funct. Mater..

[cit63] He Y., Quinn J. T. E., Lee S., Guan Y., Xu Li, Wang J., Li Y. (2017). An aromatic amine-containing polymer as an additive to ambipolar polymer semiconductor realizing unipolar N-type charge transport. Org. Electron..

[cit64] Sun H. L., Tang Y. M., Guo H., Uddin M. A., Ling S. H., Wang R. Z., Wang Y. F., Zhou X., Woo H. Y., Guo X. G. (2019). Sol. RRL.

[cit65] Sun H. L., Tang Y. M., Koh C. W., Ling S. H., Wang R. Z., Yang K., Yu J. W., Shi Y. Q., Wang Y. F., Woo H. Y., Guo X. G. (2019). Adv. Mater..

[cit66] Feng K., Zhang X., Wu Z., Shi Y., Su M., Yang K., Wang Y., Sun H., Min J., Zhang Y., Cheng X., Han Y., Guo X. (2019). Fluorine-Substituted Dithienylbenzodiimide-Based N-Type Polymer Semiconductors for Organic Thin-Film Transistors. ACS Appl. Mater. Interfaces.

[cit67] Shi Y. Q., Tang Y. M., Yang K., Qin M. C., Wang Y., Sun H. L., Su M. Y., Lu X. H., Zhou M., Guo X. G. (2019). Thiazolothienyl imide-based wide bandgap copolymers for efficient polymer solar cells. J. Mater. Chem. C.

[cit68] Lei T., Wang J. Y., Pei J. (2014). Roles of flexible chains in organic semiconducting materials. Chem. Mater..

[cit69] Lei T., Cao Y., Zhou X., Peng Y., Bian J., Pei J. (2012). Systematic investigation of isoindigo-based polymeric field-effect transistors: design strategy and impact of polymer symmetry and backbone curvature. Chem. Mater..

[cit70] Shi Y. Q., Wang Y. F., Guo X. G. (2019). Recent Progress of Imide-functionalized N-type Polymer Semiconductors. Acta Polym. Sin..

[cit71] Kim H. I., Kim M.-J., Choi K., Lim C., Kim Y.-H., Kwon S.-K., Park T. (2018). Improving the Performance and Stability of Inverted Planar Flexible Perovskite Solar Cells Employing a Novel NDI-Based Polymer as the Electron Transport Layer. Adv. Energy Mater..

[cit72] Shi Y. Q., Guo H., Huang J. C., Zhang X. H., Wu Z., Yang K., Zhang Y. J., Woo H. Y., Ortiz R. P., Zhou M., Guo X. G. (2020). Distannylated bithiophene imide: enabling high-performance N-Type polymer semiconductors with an acceptor-acceptor backbone. Angew. Chem., Int. Ed..

[cit73] Shi Y. Q., Chen W., Wu Z., Wang Y., Sun W. P., Yang K., Tang Y. M., Woo H. Y., Zhou M., Djurǐsíc A. B., He H. B., Guo X. G. (2020). J. Mater. Chem. A.

